# Supplementary material for: The causal relationship between the human gut microbiota and pyogenic arthritis: a Mendelian randomization study
Source: Front Cell Infect Microbiol. 2024 Nov 26;14:1452480. doi: 10.3389/fcimb.2024.1452480 (PMC11629706; doi:10.3389/fcimb.2024.1452480)
Supplement: Supplementary file 1 [file DataSheet1.zip › mendelian test/ebi-a-GCST90027491.csv_leave_one_out.pdf]

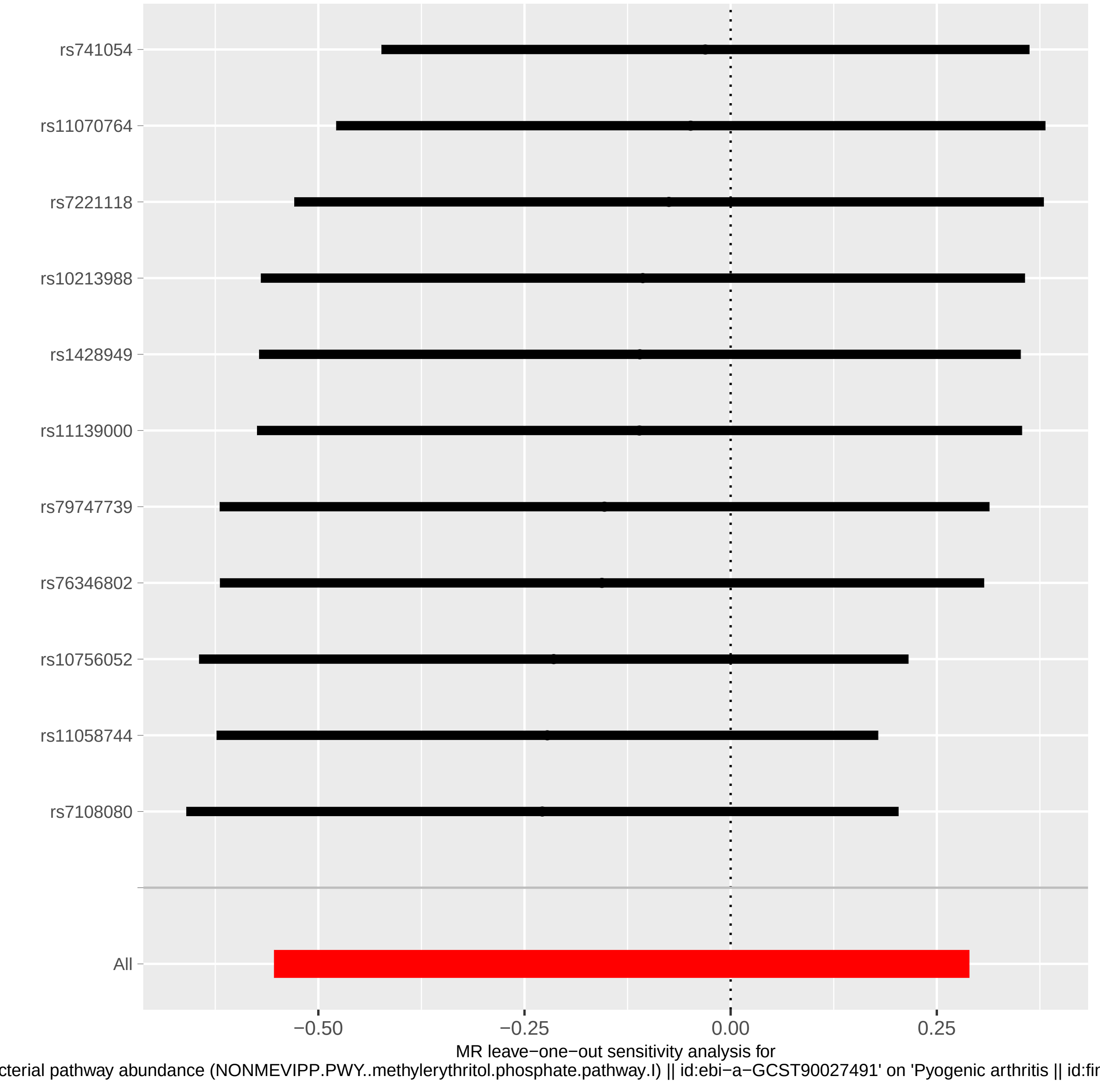
rs741054
rs11070764
rs7221118
rs10213988
rs1428949
rs11139000
rs79747739
rs76346802
rs10756052
rs11058744
rs7108080
All
−0.50
−0.25
0.00
0.25
MR leave−one−out sensitivity analysis for
cterial pathway abundance (NONMEVIPP.PWY..methylerythritol.phosphate.pathway.I) || id:ebi−a−GCST90027491' on 'Pyogenic arthritis || id:fir
